# Radiomodulation in Mixed, Complex Cancer Pain by Triple Target Irradiation in the Brain: A Preliminary Experience

**DOI:** 10.7759/cureus.25430

**Published:** 2022-05-28

**Authors:** Eduardo E Lovo, Alejandra Moreira, Claudia Cruz, Gabriel Carvajal, Kaory C Barahona, Victor Caceros, Alejandro Blanco, Ricardo Mejias, Eduardo Alho, Tatiana Soto

**Affiliations:** 1 Radiosurgery, International Cancer Center, Diagnostic Hospital, San Salvador, SLV; 2 Neurosurgery, International Cancer Center, Diagnostic Hospital, San Salvador, SLV; 3 Anesthesia and Pain Management, International Cancer Center, Diagnostic Hospital, San Salvador, SLV; 4 Pain Management, National Center for Pain Management and Palliative Care, San Jose, CRI; 5 Radiation Oncology, International Cancer Center, Diagnostic Hospital, San Salvador, SLV; 6 Radiosurgery, Robotic Radiosurgery Center, San Jose, CRI; 7 Physics, Robotic Radiosurgery Center, San Jose, CRI; 8 Functional Neurosurgery, University of Sao Paulo Medical School, Sao Paulo, BRA; 9 Radiation, Robotic Radiosurgery Center, San Jose, CRI

**Keywords:** palliative care, opioid, cancer, pain, radiosurgery, radiomodulation

## Abstract

Introduction

Up to 30% of terminally ill cancer patients experiencing intense pain might be refractory to opioid treatment. Complex cancer pain can be a mixture of somatic, visceral, and neuropathic pain with few or no effective alternatives to ameliorate pain. Radiosurgery to treat refractory pain in cancer has been reported with different degrees of success. Radiomodulation in pain could be defined as a fast (<72 h), substantial (>50%) pain relief by focal irradiation to a peripheric, and/or central mediated pain circuitry. Based on our previous experience, mixed, refractory cancer pain is usually unresponsive to single target irradiation of the hypophysis. We treated three patients using a multi-target approach.

Methods

Three terminally ill oncological patients experiencing refractory, complex, mixed pain from bone, abdomen, thorax, and brachial plexus were treated with triple target irradiation which consisted of irradiating with a maximum dose (Dmax) of 90 Gy to the hypophysis using either an 8 mm collimator with gamma ray (Infini) (Shenzhen, China: Masep Medical Company) or a 7.5 circular collimator with Cyberknife (Sunnyvale, CA: Accuray Inc.), the other two targets were the mesial structures of the thalamus bilaterally using a 4 mm collimator with Infini and the 5 mm circular collimator with CK delivering 90 Gy Dmax to each region. Patients had a VAS of 10 despite the best medical treatment.

A correlation was made between the 45 Gy and 20 Gy isodose curves of the two different technologies to the Morel stereotactic atlas of the thalamus and basal ganglia for further understanding of dose distribution reconstructions in accordance with the São Paulo-Würzburg atlas of the Human Brain Project were performed. Lastly, a scoping review of the literature regarding radiosurgery for oncological pain was performed.

Results

Radiomodulation effect was achieved in all patients; case 1 had a VAS of five at 72 h, three at 15 days, and three at the time of death (21 days after treatment). Case 2 had a VAS of six at 72 h, five at 15 days, and four at the time of death (29 days after treatment). Case 3 had a VAS of five at 72 h, six at 15 days, and six at the time of death (30 days). Morphine rescues for cases 1 and 2 were reduced to 84%, and 70% for case 3. Overall, there were no adverse effects to treatment although excessive sleepiness was reported by one patient. After reading the title and abstract, only 14 studies remained eligible for full-text evaluation, and only nine studies met inclusion criteria after full-text reading. For most reports (seven), the target was the hypophysis and in two reports, the target was the thalamus either with single or bilateral irradiation.

Conclusions

In complex, for refractory oncological pain of mixed nature (nociceptive, neuropathic, and visceral), very few, if any, treatment alternatives are currently available. We provide a small proof of concept that multitarget intracranial radiosurgery might be effective in ameliorating pain in this population. The doses administered per target are the lowest that have shown effectiveness thus far, a different strategy might be needed as opposed to single target “large” dose approach that has been tried in the past for complex mixed refractory oncological pain. By no means, in our experience, these treatments traduce in elimination of pain, clinical results might make pain to be more bearable and respond better to pain medication.

## Introduction

Up to 30% of terminally ill cancer patients experiencing intense pain might be refractory to opioid treatment administered systemically or intrathecally (10%) and thus all other conventional methods to find relief [1-3]. Current pain management with opioids especially at the high amounts that might be needed as breakthrough, or rescue doses is associated with important secondary effects that include gastrointestinal symptoms, respiratory depression, and lethargy. Since its origin, radiosurgery was conceptualized and has been used for pain relief in non-oncological and oncological pain with various success rates, the classical approaches have been single target and a few dual target irradiation using ablative doses (>130 Gy) to achieve different degrees of pain relief [4-14]. For bone-derived pain, mainly in hormone-mediated cancers such as breast and prostate, a high dose of radiation to the hypophysis has been described as successful in most patients (>70%) [7-11,14]. In our experience, this approach may not be as effective in abdominal or pelvic pain from histology such as ovarian and pancreatic cancer or neuropathic pain derived from brachial plexus involvement in Pancoast syndrome [14]. Radiomodulation in pain could be defined as a fast (<72 h), substantial (>50%) pain relief by focal irradiation to a peripheric and/or central mediated pain circuitry, ideally with a sub ablative dose, although the clinical importance of lesser doses in oncological pain in terminally ill patients remains to be proven.

Recently our group published a novel, dual-target approach for pain crisis management in trigeminal neuralgia where all other approaches had failed, the main objective of this approach was to consistently provoke radiomodulation. The dual targets irradiated were the affected trigeminal nerve with 90 Gy and the contralateral structures of the medial thalamus, which encompass in part the perifascicular complex (PFc) and centromedian nucleus (CM) with 140 Gy, a dose considered ablative. In this communication, the last patient treated with success was using a dose of 80 Gy to the nerve and 120 Gy to the thalamus, in this series radiomodulation was achieved in 100% of the population leading to pain relief of at least 50% in less than 72 h [15]. More recently, patients have been treated with 80 Gy to the nerve and 90 Gy to the thalamus with the same success [unpublished data], demonstrating that lower doses could be effective when multi-target radiosurgery is performed for pain.

We present preliminary clinical evidence in three patients with refractory pain that were treated with triple target irradiation with what might be considered low doses of radiation in comparison to the more traditional single target, ablative dose approach.

## Materials and methods

From January to March 2022, three terminally ill oncological patients experiencing mixed pain from bone, abdomen, thorax, and brachial plexus, refractory to opioids were treated with triple target irradiation. This approach consisted of irradiating with a maximum dose (Dmax) of 90 Gy to the hypophysis using either an 8 mm collimator with gamma ray (Infini) (Shenzhen, China: Masep Medical Company) or a 7.5 circular collimator with Cyberknife (CK) (Sunnyvale, CA: Accuray Inc.), the other two targets were the mesial structures of the thalamus bilaterally using a 4 mm collimator with Infini and the 5 mm circular collimator with CK delivering 90 Gy Dmax to each region. These patients were referred by palliative and pain medicine specialists as they were deemed refractory to all other treatments available and despite best efforts, their perceived pain scale was 10 in the visual analogue scale (VAS). Their cases were discussed and approved by a multidisciplinary team of algologists, radiation oncologists, and neurosurgeons. All patients and caregivers signed informed written consent. This treatment protocol was approved by the International Cancer Center Group ethical committee board. We correlate the isodose curves of 45 Gy and 20 Gy of two different technologies to the Morel stereotactic atlas of the thalamus and basal ganglia, for further understanding of dose distribution three-dimensional reconstructions in accordance with the São Paulo-Würzburg atlas of the Human Brain Project were performed, lastly, we carried out a review of the literature of radiosurgery for oncological pain.

Radiosurgical technique

**Figure 1:**
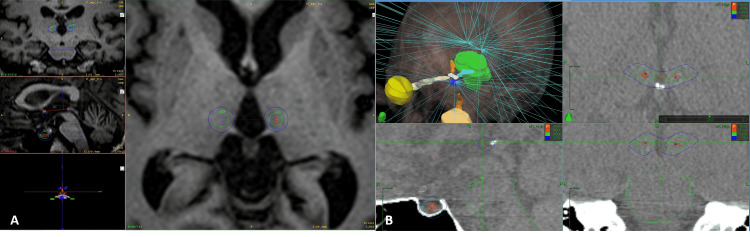
Treatment plans for the Infini (Shenzhen, China: Masep Medical Company) and the Cyberknife (CK) (Sunnyvale, CA: Accuray Inc.). (A) Infini plan showing the three-target irradiation, from left to right, coronal, axial, and sagittal view, the 45 Gy isodose line is represented in green and the outer blue circle represents the 20 Gy isodose line. (B) Cyberknife plan with the three-target irradiation, from left to right and from top to bottom: three-dimensional reconstruction of the pencil beams and the organs at risk, axial, sagittal, and coronal views, the red inner circles represent the 80 Gy isodose line, green isodose line represents the 45 Gy, and the most outer line the 20 Gy isodose curve.

On the day of the procedure, under local anesthesia and slight sedation a stereotactic frame was placed by a neurosurgeon with the aid of ear bars searching for a frame parallelism to the anterior and posterior commissure line or the intercommissural line (ICL) for the Infini. For CK a thermoplastic mask was configured seeking the ICL parallelism with the CT lasers. For Inifni an MRI acquisition was performed with a 1.5-Tesla Avanto (Erlangen, Germany: Siemens Corporation) of the full head, a T1 and T2 constructive interference in steady state of 1 mm slice thickness with no spacing covering the thalamus until the superior border of the corpus callosum; for both patients treated with CK they were implanted with intrathecal or peripheral drug delivery pumps that made MRI studies not possible thus CT of 0.625 mm thickness with no spacing was done of the whole head including the jaw. Images were later transferred to the treatment planning stations (Superplan for Infini and Multiplan for CK). For treatment planning, the anterior commissure (AC) and posterior commissure (PC) were identified in the axial plane, then images were transformed into a sagittal plane using fusion tools and the ICL was drawn. The distance from the PC was taken anteriorly along the ICL (usually 4 mm); this measure was tagged as Y. A 90º angle was traced from the PC to the Y along the ICL and Z was determined usually 4 mm above the ICL. Images were then reoriented to axial views, and X coordinates were 4 to 5 mm lateral from the thalamic border on each side. Using a 4 mm collimator with Infini and a 5 mm cone with CK, a single shot was placed in each region of the thalamus prescribing 90 Gy as Dmax bilaterally, the 20 Gy isodose line was plotted as a possible “area of influence” of radiation (Figure 1).

For the hypophysis target efforts were made to identify the neurohypophysis and define this as the isocenter of the shoot, using an 8 mm with Infini and a 7.5 mm collimator with CK 90 Gy Dmax was delivered, the complete optic pathway and brainstem were defined as an organ at risk so that in any case the dose would not be superior to 8 Gy in the optic pathway or 15 Gy at a focal point in the brainstem. For the CK the plan was isocentric with 90 Gy to 97% using a short path and Monte Carlo algorithm at high resolution for dose calculations, as it is standard for this machine 6D skull tracking was used.

Patients were contacted 24, 48, 72, and 96 h after treatment and every week thereafter until the time of death. Radiomodulation effect was defined as a prompt relief of pain (reduction in VAS of at least 50%) specifically within the first 72 h after stereotactic radiosurgery (SRS). Morphine rescue as an additional parenteral administration of morphine over the basal needs of pain medications was considered a more objective indicator of pain relief. Patients were asked to level their pain during every follow-up call and to report any increase in pain not responding to their usual medication as well as the amount of morphine rescues required per day.

Atlas correlation

**Figure 2:**
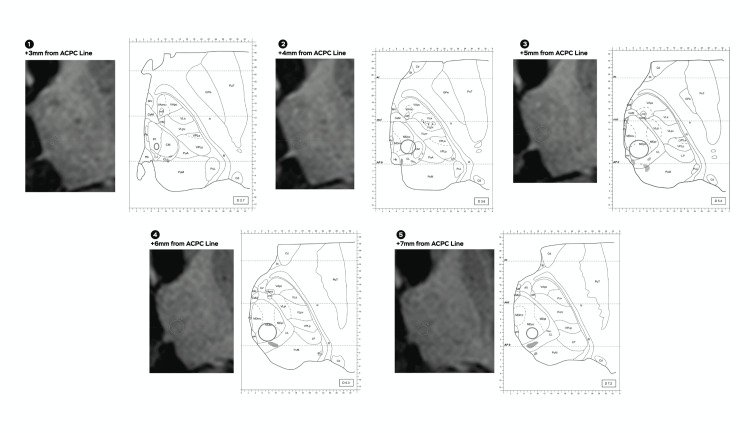
Morel atlas correlation shows a 4 mm collimator shoot and 45 Gy isodose line. An approximate correlation to the nearest millimeter in proximity to the Morel atlas shows a 4 mm collimator shoot and the 45 Gy isodose line, numbers from one to five dissect different distances regarding the anterior commissure and the posterior commissure line (ACPC line). Pf: parafascicular; CM: centromedian; MDpc: medial dorsal parvocellular

**Figure 3:**
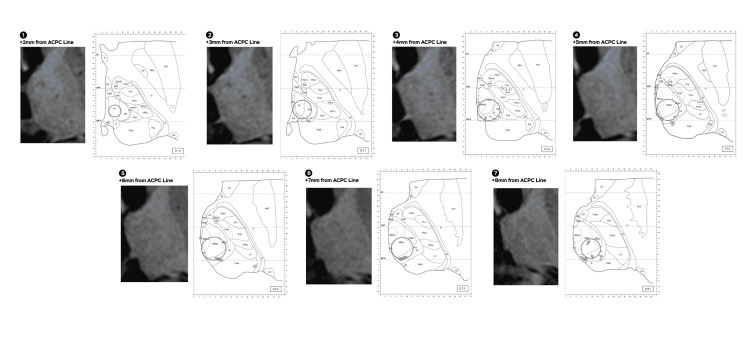
Morel atlas correlation shows a 4 mm collimator shoot and 20 Gy isodose line. An approximate correlation to the nearest millimeter in proximity to the Morel atlas shows a 4 mm collimator shoot and 20 Gy isodose line, numbers from one to seven dissect different distances with regard to the anterior commissure and the posterior commissure line (ACPC line). Pf: parafascicular; CM: centromedian; MDpc: medial dorsal parvocellular

**Figure 4:**
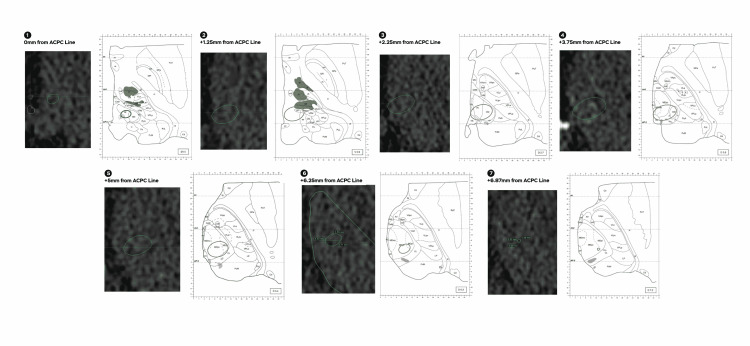
Morel atlas correlation shows a Cyberknife (Sunnyvale, CA: Accuray Inc.) 5 mm collimator and 45 Gy isodose line. An approximate correlation to the nearest millimeter in proximity to the Morel atlas shows a 5 mm collimator shoot and 45 Gy isodose line, numbers from one to seven dissect different distances with regard to the anterior commissure and the posterior commissure line (ACPC line). Pf: parafascicular; CM: centromedian; MDpc: medial dorsal parvocellular; MDmc: medial dorsal magnocellular; MDpl: mediadorsal paralaminar

**Figure 5:**
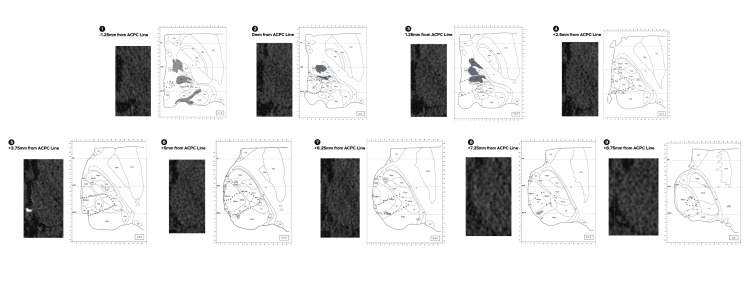
Morel atlas correlation shows a Cyberknife (Sunnyvale, CA: Accuray Inc.) 5 mm collimator and 20 Gy isodose line. An approximate correlation to the nearest millimeter in proximity to the Morel atlas shows of a 5 mm collimator shoot and 20 Gy isodose line, numbers from one to nine dissect different distances with regard to the anterior commissure and the posterior commissure line (ACPC line). Pf: parafascicular; CM: centromedian; MDpc: medial dorsal parvocellular; MDmc: medial dorsal magnocellular; MDpl: mediadorsal paralaminar

For Infini and CK, isodose correlation was approximated as much as possible to the Anne Morel stereotactic atlas of the thalamus and basal ganglia (Morel atlas) by measuring the anterior to posterior diameter of the 45 and 20 Gy isodose line as well as its medial to lateral diameter, distance from the thalamic border (TB) was considered in reference to the isocenter of the shoot that was 4 mm anterior to the posterior commissure, for the CK due to its irregular shape the maximal distance from the TB to the 45 and 20 Gy isodose lines was considered as well as the distance from the TB to the most anterior and posterior point and extrapolated to the atlas. With regards to cephalocaudal (Z) direction from the anterior commissure and the posterior commissure (ACPC) line, Infini was taken every 1 mm and for CK every 1.25 mm and correlated to the closest image on the atlas (Figures 2-5).

**Figure 6:**
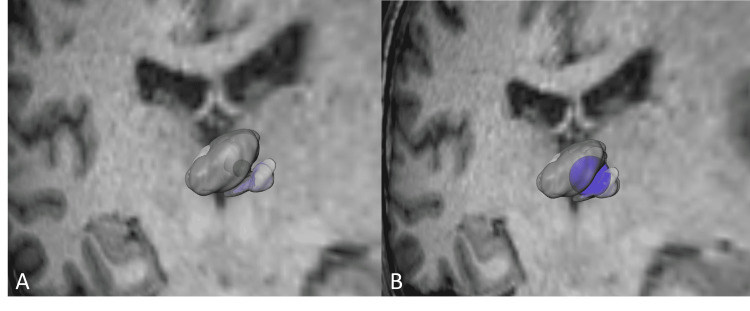
Anterolateral three-dimensional view of Infini (Shenzhen, China: Masep Medical Company) 4 mm shoot. (A) An anterolateral reconstruction of the 45 Gy isodose line represented by the small green circle inside the centromedian nucleus represented in light purple, the perifascicular complex (PFc) in dark purple, and the medial thalamic group in shades of green. (B) An anterolateral reconstruction of the 20 Gy isodose line represented in dark blue inside the centromedian nucleus represented in light purple, the PFc in dark purple, and the medial thalamic group in shades of green.

**Figure 7:**
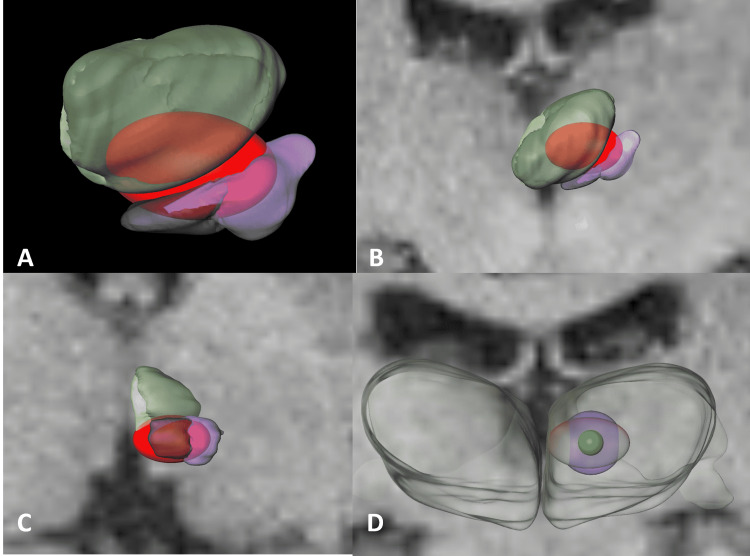
Different views of a reconstruction of the Cyberknife 5 mm, 45 Gy isodose line. (A) Anterior three-dimensional reconstruction view of the prafascicular complex in dark purple, the centromedian nucleus in light purple, and the medial thalamic group in light green, inside in red the 45 Gy isodose line of the Cyberknife. (B and C) Anterior and ventral view for the 45 Gy isodose line in red. (D) An anterior view comparing the 45 Gy isodose line of the Infini (Shenzhen, China: Masep Medical Company) in green, the 20 Gy in light blue, and the oval, light red corresponds to the 45 Gy isodose line for the Cyberknife (Sunnyvale, CA: Accuray Inc.).

For an additional validation and greater three-dimensional comprehension of the areas affected by radiation, thalamic nuclei were segmented according to Hassler's cytoarchitectonic criteria in histological plates and registered into a post-mortem MRI and normalized to a stereotactic space (ICBM 152 MNI space) as part of the São Paulo-Würzburg atlas of the Human Brain Project. The average coordinates of the isodose correspond to the 45 Gy and the 20 Gy isodose line for the Infini and the 45 Gy isodose line for the CK were centered and plotted into the histological atlas to illustrate the structures affected by radiation, the 20 Gy isodose line for CK could not be accurately reconstructed due to its irregularities in comparison to the Infini (Figures 6, 7).

Review of literature

**Figure 8:**
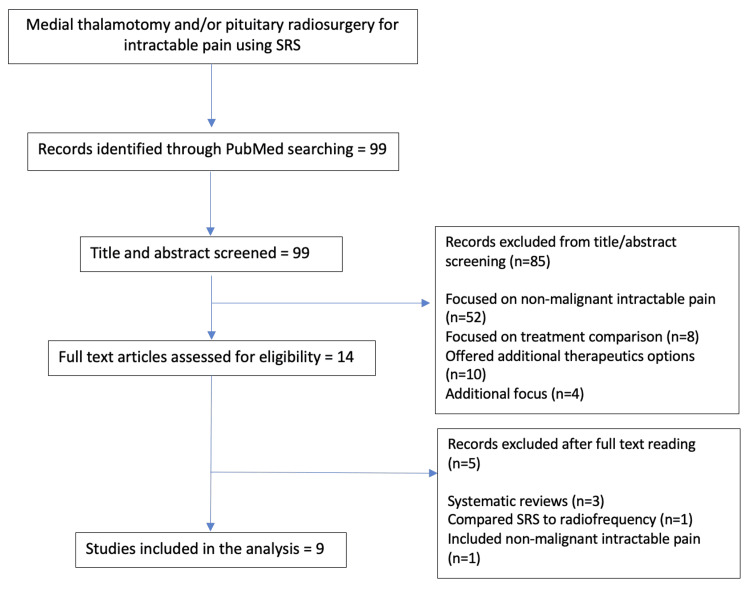
Literature review process.

Lastly, we carried out a review of the existing literature to date regarding the use of stereotactic radiosurgery for the treatment of oncologic intractable pain. PubMed was the primary database for electronic article searching using the medical subject headings (MeSH) terms “radiosurgery” AND “intractable pain”; there was no restriction on language or publication date; publication dates ranged from 1970 to 2022. Peer-reviewed articles reporting clinical outcomes and containing follow-up information regarding the safety of SRS for oncologic pain targeting either the pituitary gland and/or thalamus were considered inclusion criteria; precise target definition was also an important aspect to consider. Cases where radiosurgery was performed due to chronic pain originating from trigeminal neuralgia or other cranial nerve neuralgia and where SRS was used for other conditions than pain were excluded. Follow-up time was not a criterion for exclusion given the prognosis of the disease in patients included in this series. After title and abstract reading, only 14 studies remained eligible for full-text evaluation and after full-text reading, only nine studies met with inclusion criteria (Figure 8). Table 1 summarizes our findings regarding pituitary and/or thalamic radiosurgery for the management of oncologic intractable pain.

**Table 1:** Review of literature on radiosurgery in oncologic pain. SRS: stereotactic radiosurgery; CM: centromedian; Pfc: parafascicular complex; VAS: visual analogue scale

Study	Number of patients	Cause of pain	Radiosurgical target	Dose (Gy)	Follow-up (months)	Results	Side effects
SRS Thalamotomy							
Leksell (1972) [4]	25	Chronic pain due to malignant disease	CM-Pfc. 25 opposite side to where the most severe pain was felt + 7 bilateral lesions	200-250	1-12	Therapeutic effect of irradiation was apparent 2-3 weeks postoperatively. Six patients were virtually free of pain and remained so for over a year. Four patients experienced moderate decrease in pain. In 9 there was only a slight effect and in 6 patients no amelioration was obtained.	No surgical complications. Two patients who became free of pain after 3 weeks displayed hemianesthesia about 2 months later.
Steiner (1980) [5]	50	Chronic pain due to malignant disease	CM-Pfc. 24 unilateral and 26 bilateral lesions	140-250	ND	Eight patients experienced good pain relief, 18 had moderate relief and in 24 the procedure did not significantly influence the pain	Weakness of upward gaze, vertigo, hemianesthesia, paresthesia, and hypesthesia.
Pituitary radiosurgery							
Backlund et al. (1972) [6]	8	Bone metastasis from breast carcinoma	Adenohypophysis	200-250	4-9	Evaluation done on only 4 patients, who had considerable pain relief and improvement.	Frequent episodes of diabetes insipidus
Liscák and Vladyka (1998) [7]	1	Bone metastasis from breast carcinoma	Pituitary gland	150	26	Complete pain relief lasting 24 months after SRS	At long-term follow-up, decreased serum cortisol and requiring hormonal replacement after SRS
Hayashi et al. (2002) [8]	9	Bone metastasis (different origins)	Junction between pituitary gland and stalk	160	1-24	From the 9 patients included, all had complete pain relief and not use of permanent medication was required	None
Hayashi et al. (2004) [9]	10	Bone metastasis (different origins)	Junction between pituitary gland and stalk	160	1-6	8 patients had complete pain relief (with or without morphine use) and 2 patients had considerable pain relief.	None
Kwon et al. (2004) [10]	7	Metastasis (different origins)	Junction between pituitary gland and stalk	150-160	1-13	Pain relief was documented in 5 patients (71%) and 2 patients with pain recurrence had less intense pain after SRS. For analgesic use, a reduction of 19% was documented. Overall, 86% of patients were satisfied with the treatment received.	Hormonal abnormalities in one patient with pre-existing diabetes insipidus and hypopituitarism
Lovo et al. (2019) [11]	10	Metastasis (different origins)	Neurohypophysis	150	1-12	Patients reported between absent to pain adequately controlled with medication one month after SRS. In 6 patients, a 25% reduction in analgesic use was documented. Long-term follow-up was seen in one patient.	None
Golanov et al. (2020) [16]	1	Lung and liver metastasis from pancreatic cancer	Junction between pituitary gland and stalk	150	1	The fifth day after treatment was the one showing maximum analgesic effect and the effect was permanent after that. Significant reduction in analgesic use was also documented and an improvement in patients’ quality of life.	None
Pituitary + thalamic radiosurgery							
Current series (2022)	3	Metastasis (different origins)	CM-Pfc complex bilaterally and neurohypophysis	90	21 days 1st patient, 29 days 2nd patient, and 30 days 3rd patient	For an average of 26 days of follow-up among the three patients, VAS average for the first patient was 3, 4 for the second patient, and 6 for the third patient. As for morphine rescue, the first patient reduced its use by around 85%, 80% for the second patient, and 70% for the third patient.	One patient reported being more sleepy than usual after SRS

## Results

Case 1

The first patient was a 65-year-old Latin male with known hypertension and benign prostatic hyperplasia. The patient was being studied for an unknown metastatic disease that proved to be a pulmonary adenocarcinoma. When the patient was referred to our center in February 2022, he presented with thoracic and lumbar metastatic pain, as well as pain in the hips and lower extremities, he could not adopt a supine position since November 2021 due to intolerable pain. Regarding previous therapeutic interventions, he had gone through radiofrequency (thoracic T10, T11, T12 and lumbar L3, L4, L5, S1) bilaterally, T11 vertebroplasty due to a pathologic fracture as well as stereotactic body radiotherapy (SBRT) with a dose of 24 Gy in three sessions (8 Gy per fraction) but was considered non-effective in either ameliorating pain or decreasing the amount of medication used. Baseline treatment for pain included extended-release morphine 30 mg every 8 h, paracetamol 750 mg every 8 h, ketorolac, and rescue morphine up to 12 times a day. Daily pain reported was VAS of 10 and after rescue morphine, he reached VAS of 3 but the effect only lasted between 30 minutes and 1 h.

Case 2

The second patient was an 18-year-old Latin female with a relapsing Ewing sarcoma. She went through surgery for tumor resection in her left hemithorax along with chemotherapy and adjuvant radiotherapy (3D technique) to the tumor resection site receiving 54 Gy in 27 fractions. A second radiotherapeutic session of 8 Gy/1 fraction at a supra and infraclavicular level was delivered in April 2021 with a palliative intention due to tumor recurrence and increasing amounts of pain. In November 2021, a third radiotherapeutic session using lattice technique was given to the recurring left hemithorax mass, and in this case, two targets were considered: planning target volume (PTV)1 (whole mass) receiving 9 Gy/3 fractions and PTV2 (mass vertex) receiving 24 Gy/3 fractions; only partial relief of pain was achieved. During progression, the patient was referred to our center because of increasing pain in her shoulder, left arm, and left chest wall due to extensive intrathoracic tumor growth and malignant brachial plexopathy.

Previous pain treatments included multiple brachial plexus blocks with local anesthetic, steroids, botulinum toxin, and considering multifocal pain distribution she had a tunneled continuous interscalene brachial plexus and an epidural catheter at T5 delivering local anesthetic. The patient required a continuous IV morphine infusion (285 mg daily), 15 mg boluses as needed every 2 h, 1800 mg daily gabapentin, and venlafaxine 75 mg. She was considered unfit for implantation of an intrathecal drug delivery system. Despite multimodal analgesia, the patient had a VAS of 10 and continued need of rescue morphine to achieve a VAS of 5.

Case 3

The third patient was a 66-year-old Latin male with stage IV pancreatic adenocarcinoma metastatic to liver, peritoneum, and bone. In June 2021, he received palliative radiotherapy to the dorsal spine (25 Gy/5 fractions), but response to treatment was only partial. The patient was referred to our center in March 2022 due to refractory oncologic pain presenting at an abdominal, thoracic, and lumbar level. Baseline pain treatment for this patient included an intrathecal pump (Synchromed II; Minneapolis, MN: Medtronic) delivering morphine 4.6 mg/24 h and bupivacaine 1.5 mg/24 h, 100 ug/h transdermal fentanyl, and intrathecal morphine rescue every 2 h through a patient controller (average of 12 rescues per day). A previous celiac plexus neurolysis was performed but deemed unsuccessful.

Treatment outcomes

**Figure 9:**
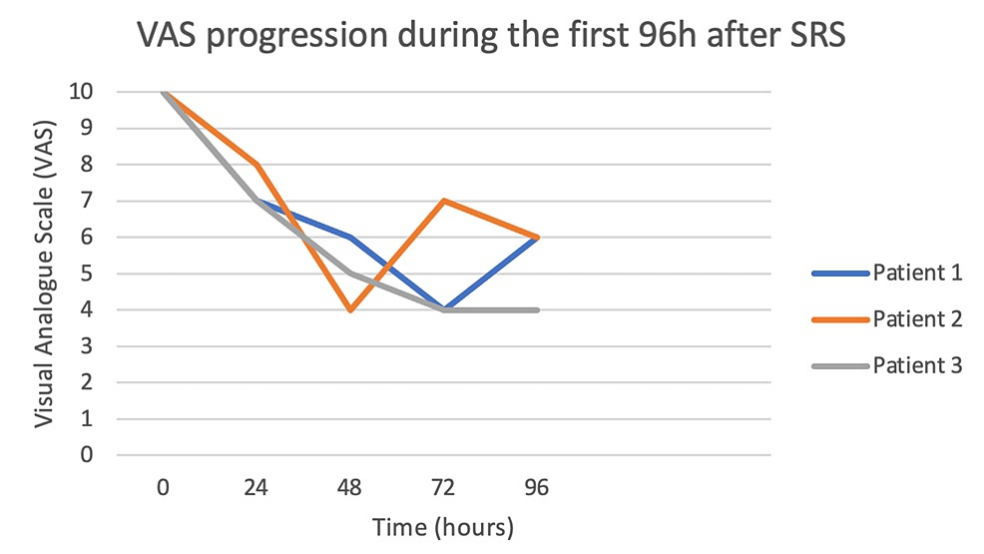
Pain reduction measured with the visual analogue scale after 96 h posttreatment. SRS: stereotactic radiosurgery

**Figure 10:**
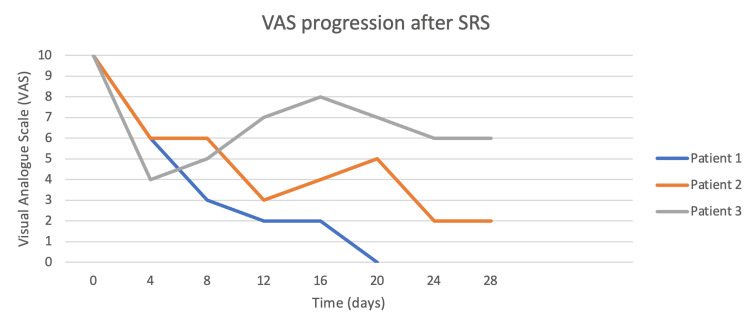
Pain reduction measured with the visual analogue scale after 96 h until death. SRS: stereotactic radiosurgery

Patient 1 was followed at 24, 48, 72, and 96 h after treatment and every week thereafter. At 24 h, he reported a VAS of 7 and required only three morphine rescues, at 72 h his VAS had decreased to 4 and required only two morphine rescues (only 16% of requirement). Six days after treatment the patient had pulmonary thromboembolism, which required hospitalization; during his hospital stay, he tolerated a supine position at 30 degrees without any sedation or morphine rescues. The blue line shows the VAS progression of patient 1 during the first 96 h (Figure 9) and after treatment and until his death (Figure 10).

**Figure 11:**
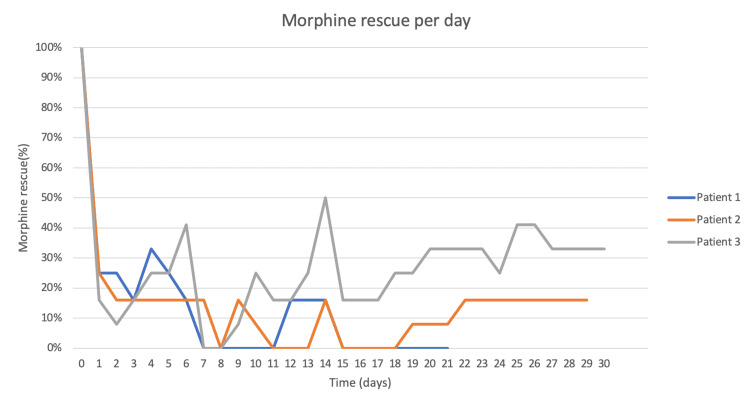
Morphine rescue per day after treatment until the time of death.

Figure 11 shows the number of rescues needed per day after SRS. During these 21 days, his VAS varied between 0 and 5, and his pain responded to baseline treatment, decreasing the number of morphine rescues he usually required; from 12 morphine rescues in a day, he was then able to spend a day without any rescue requirements to reduce pain (84% reduction in morphine rescues).

Patient 2 is represented as the orange line in Figures 9-11. At 24-h follow-up, her VAS was 8 and required only three morphine rescues (25%). Forty-eight hours after treatment, her VAS had reached 4 and only 16% of her basal morphine rescues were required. During her follow-up, the patient reported up to four days without any need for morphine rescues (0%) with only baseline treatment ameliorating her pain; other days she reported pain crisis, reaching VAS of 7 but responded to baseline treatment or required only small amounts of morphine rescues (16%). The patient died 29 days after treatment due to primary disease progression and an allergic reaction to chemotherapy; her need for morphine rescue was reduced by 84% during this period.

Patient 3 is represented by the gray line in Figures 9-11. Twenty-four hours after SRS, the patient reported a VAS of 7 requiring only two morphine rescues. At 72-hours, he had a VAS of 4 and needed only one morphine rescue. The first eight days after SRS, VAS varied between 3 and 6 and reported feeling more sleepy than usual. At his next follow-up, his VAS increased between 6 and 9, but his morphine needs were between 16% and 41%, never requiring the initial dose to manage the pain crises. VAS average for this patient in his 30 days of follow-up was 6 and during this time his morphine rescue requirements decreased up to 70%. Overall there were no adverse effects to treatment although excessive sleepiness was reported by patient 3.

## Discussion

The rationale for choosing a multiple target approach to these complex cases was that we had failed in similar cases before when we just irradiated the hypophysis [14]. Irradiating the thalamus bilaterally might have worked alone but published data at higher doses was not effective in the majority of patients [6]. Also, in our experience, irradiating this area alone is that the effects could be short-lived and be as brief as only one week as seen in non-oncological pain [12]. Thus, a triple target strategy with substantially lower doses seemed to be safe and hopefully a successful clinically approach.

Refractory oncological pain imposes a high burden on patients and caregivers worldwide, the earlier reports of radiosurgery to the hypophysis have shown high grades of quick pain relief practically in all series, nevertheless, the experience is extremely limited, and the numbers are very small as can be appreciated in the scoping review of literature of the last 50 years. From the clinical point of view, this might be the preferred approach for pain derived from extensive metastases to the bone mainly in breast and prostate cancer patients, modern doses with a single target approach have been in the magnitudes of 140-150 Gy and most series do not report clinical adverse endocrine events such as diabetes insipidus. In our initial experience, it was clear that this treatment alternative was highly effective in reducing pain but by no means does it eliminate pain and in most cases, flairs of pain are to be expected especially days or weeks close to the death of the patients [14]. As in all complex pain, a multiple drug-intervention approach is needed, and active surveillance and assistance from pain medication when necessary. Our current understanding of the underlying mechanisms of pain relief when a high dose of radiation is delivered to the hypophysis is merely speculative but the redirection of oxytocin is suspected, thus a possible radio-endocrine-modulatory effect as opposed to a radiomodulatory effect can be obtained by irradiation of a peripheric nerve or a central pain relay center, where no hormones are produced or stored [17].

For medial thalamus irradiation for oncological pain, the evidence is even more inconclusive, in our initial series, we treated only one terminally ill oncological patient that experienced pain alleviation from an advanced head and neck cancer [12]. This anatomical area has been better studied as a central pain relay center, the main area of irradiation being the PFc and CM are believed to serve a "gate control" function, propagating only salient noxious stimuli and suppressing certain other stimuli. Also, the CM serves as a relay for the paleospinothalamic tract as some of its afferents are to the cingulum and fornix, it is alleged that this mediates and processes the affective component of pain [18]. Although the least amount of dose capable of radiomodulation in humans is unknown, doses as low as 13 Gy have been reported as capable of inducing cessation of epileptic seizures [19]. Animal models show that in the immediate phase after radiosurgery there might be a lesser focal consumption of glucose, in the median term (days) lesser dose (10-40 Gy) produces an excitatory effect, and higher doses of 50-120 Gy produce an inhibitory effect [20]. Thus, we hypothesize that the area of influence between 12 and 20 Gy isodose might also be important for radiomodulation purposes.

As can be seen in the correlation with the coordinates that we used in the Morel atlas, there are also important differences in areas covered by the 45 Gy isodose line but become more relevant in the 20 Gy isodose line when a Dmax of 90 Gy is delivered either by gamma ray (Knife), Infini technology as opposed to Cyberknife, the physical reasons are mainly due to a different size collimator, a different source-axis distance (SAD), and energy levels that favor gamma ray for a stepper dose fall off. When Infini is used the 45 Gy isodose line covers small parts of the PFc, CM, and the medial dorsal parvocellular (MDpc), with regards to the 20 Gy isodose line we can see a much larger involvement of the PFc and CM as well as the MDpc and the medial dorsal magnocellular (MDmc) and the mediodorsal paralaminar (MDpl) portions of the MD nucleus. For Cyberknife and the 45 Gy isodose line, there is much wider coverage of the PFc and CM as well as the MDpc, MDcm, and MDpl. When the 20 Gy isodose line is concerned most of the PFc and CM are covered also the majority of the MD (mediodorsal) nucleus in its three components. We also can expect involvement of the thalamic nucleus in the lateral group such as the ventroposterolateral (VPL) and the ventroposteromedial (VPM) that are considered part of the spinothalamocortical pathway, these are well known primary sensory nuclei that relay directly to the primary sensitive cortex [21]. The MD, especially its medial portion or the MDpc, is influenced by the 45-20 Gy isodose lines with both Gamma and Cyberknife; these portions of the MD, as well as CM, are part of the anterior nuclei limbic group and the sensorimotor/limbic also known as the bridging nuclei that would include the more medial nucleus, the PFc as well as the central and lateral parts of the MD are considered part of the limbic areas of the thalamus and thus serve a direct role in limbic-associated with affective function [21,22].

Although originally thought that doses below 130 Gy were sub necrotic, evidence in animal studies for epileptic models suggests that a high dose of radiation delivered with a gamma knife has shown a decrease of up to 55% of neuronal firing compared to the control animals, and rats receiving <50 Gy had no signs of necrosis [23,24]. Additional information suggests that in gray matter 100 Gy might be sufficient to cause necrosis and as low as 60 Gy in white matter [25]. Another study using a single dose of 40 Gy did not cause apparent necrosis in minipig's brain thus when a Dmax of 90 Gy is delivered less than a 1.5 mm diameter inside the target might receive a dose equal and superior to 80 Gy [26]. A smaller dose (i.e., 20 Gy the area of influence) and a higher dose of 40-80 Gy might slow neuronal transmission at first, while higher dose might impair neuronal activity posteriorly. The RM effect might be due to both phenomena, with an initial almost immediate decrease in neuronal transmission of pain (thus a favorable clinical outcome) and a later (possible) impairment of neuronal activity.

Recently, successful clinical evidence of SBRT irradiation of the celiac plexus for abdominal pain is emerging, dose is 25 Gy to a relatively large target around the abdominal aorta [27]. Their success rates are high which is very promising, nevertheless, it seems to take time and the patients selected were in a favorable ECOG 1 and intermediate levels of pain VAS six, which is most contrasting with the patients we report with abdominal pain. Based on neuroanatomical knowledge of main pain relay centers and scarce clinical evidence such as the one we present; it might be possible that a peripheric (celiac plexus) and bilateral irradiation to the thalamus (central) might provide a faster radiomodulation (RM) effect in patients in more delicate conditions with higher pain levels (7-10) that merit a faster pain response. There is enough evidence to understand that unilateral irradiation of the mesial structures of the thalamus with ablative dose is safe, in our three patients' bilateral irradiation with a mostly suspected sub ablative dose also seemed safe, there was one patient that reported feeling more sleepy than usual, left to neuroanatomy alone this could be the PFc bilateral inhibition as PFc is associated with sleep, change in patterns, and arousal and also the paraventricular nuclei (Pv) as it is linked and interconnected to the hypothalamic suprachiasmatic nucleus considered the pacemaker of the circadian rhythm with sleep and awake regulation [28,29]. As seen in the 20 Gy isodose line and the correlation to the Morel atlas these two areas can be, especially the latter, encompassed by significant radiation, especially with Cyberknife with the current treatment strategy. Nevertheless, under the context of advanced disease progression and medicine side effects and this being a single patient in this small patient case series, the underlying explanation is inconclusive and larger clinical evidence with this approach is necessary.

These initial case reports provide a proof of concept that the use of smaller doses of radiation in a multiple target approach for the management of complex, refractory oncological pain might potentially be useful where all other alternatives have failed. These patients exemplify diverse and complex pain syndromes emerging from different areas of the body where a single target approach might be otherwise ineffective, multiple neuronal circuitry intervention with lesser and hopefully true sub ablative doses could represent an alternative for treatment. There is animal evidence that 60 Gy or less might be “the true” sub ablative dose, further clinical case series might explore even lesser doses than the ones here reported under a multiple target tactic [26]. Although in terminally ill oncological patients we might reach a thin line between effectiveness such as that demonstrated in the classical series of irradiation of the hypophysis with ablative doses and not being effective with lesser doses, however, it seems worthwhile that an effort must be made to understand the minimal effective dose.

Despite advances in interventional pain medicine and drug delivery, there is a substantial number of patients that suffer quite dearly in their end-of-life phase, efforts are needed to find other alternatives that ameliorate their grieving and improve their quality of life.

## Conclusions

In complex, for refractory oncological pain of mixed nature (nociceptive, neuropathic, and visceral), very few, if any, treatment alternatives are currently available. We provide a proof of concept that multitarget intracranial radiosurgery might be effective in ameliorating pain in this population as expressed in the VAS and morphine rescue consumption. Further studies are needed with larger case series to demonstrate an improvement in quality of life of these patients and their family members in this critical stage in their lives.

The doses administered per target are the lowest that have shown effectiveness thus far, a different strategy was needed as opposed to single target “large” dose approach that has been tried in the past and at least in our experience has proven ineffective in mixed, refractory oncological pain. By no means, in our experience these treatment strategies either single, dual, or triple target irradiation traduce in elimination of pain in most oncological patients, clinical results have allowed an unmanageable intense pain to be more bearable and to respond better to basal pain medication.

There is very little available published evidence regarding the use of radiosurgery for the management of oncological pain, it seems that minute efforts are being made to innovate in pain management. If 'low doses' of focal radiation can effectively reduce pain significantly in a brief timespan, radiosurgery might prove to be the safest noninvasive alternative available in the management of refractory oncological pain at this moment.
